# Hepatocyte-intrinsic type I interferon signaling reprograms metabolism and reveals a novel compensatory mechanism of the tryptophan-kynurenine pathway in viral hepatitis

**DOI:** 10.1371/journal.ppat.1008973

**Published:** 2020-10-12

**Authors:** Alexander Lercher, Alexandra M. Popa, Csilla Viczenczova, Lindsay Kosack, Kristaps Klavins, Benedikt Agerer, Christiane A. Opitz, Tobias V. Lanz, Michael Platten, Andreas Bergthaler

**Affiliations:** 1 CeMM Research Center for Molecular Medicine of the Austrian Academy of Sciences, Lazarettgasse, Vienna, Austria; 2 DKTK Brain Cancer Metabolism Group, German Cancer Research Center (DKFZ), Heidelberg, Germany; 3 Neurology Clinic and National Center for Tumor Diseases, University Hospital of Heidelberg, Heidelberg, Germany; 4 Division of Immunology and Rheumatology, Department of Medicine, Stanford University School of Medicine, Stanford, CA, United States of America; 5 Department of Neurology, University of Heidelberg, Medical Faculty Mannheim, Mannheim, Germany; 6 DKTK Clinical Cooperation Unit Neuroimmunology and Brain Tumor Immunology, German Cancer Research Center (DKFZ), Heidelberg, Germany; Albany Medical College, UNITED STATES

## Abstract

The liver is a central regulator of metabolic homeostasis and serum metabolite levels. Hepatocytes are the functional units of the liver parenchyma and not only responsible for turnover of biomolecules but also act as central immune signaling platforms. Hepatotropic viruses infect liver tissue, resulting in inflammatory responses, tissue damage and hepatitis. Combining well-established *in vitro* and *in vivo* model systems with transcriptomic analyses, we show that type I interferon signaling initiates a robust antiviral immune response in hepatocytes. Strikingly, we also identify IFN-I as both, sufficient and necessary, to induce wide-spread metabolic reprogramming in hepatocytes. IFN-I specifically rewired tryptophan metabolism and induced hepatic tryptophan oxidation to kynurenine via *Tdo2*, correlating with altered concentrations of serum metabolites upon viral infection. Infected *Tdo2*-deficient animals displayed elevated serum levels of tryptophan and, unexpectedly, also vast increases in the downstream immune-suppressive metabolite kynurenine. Thus, *Tdo2*-deficiency did not result in altered serum homeostasis of the tryptophan to kynurenine ratio during infection, which seemed to be independent of hepatocyte-intrinsic compensation via the IDO-axis. These data highlight that inflammation-induced reprogramming of systemic tryptophan metabolism is tightly regulated in viral hepatitis.

## Author summary

Viral hepatitis is responsible for more than one million annual deaths worldwide and may progress to liver cirrhosis and hepatocellular carcinoma. The main metabolic cell type of the liver is the hepatocyte. In viral hepatitis, type I interferon (IFN-I) signaling rewires hepatocyte metabolism and serum metabolites to shape disease pathophysiology – an immune-regulatory circuit that might be therapeutically exploited. Here, we show that hepatocyte-intrinsic antiviral IFN-I signaling is both necessary and sufficient to induce wide-spread metabolic changes in hepatocytes. We identify a IFN-I-mediated induction of the hepatic kynurenine pathway via the rate-limiting and liver-specific enzyme TDO2, which controls serum homeostasis of tryptophan by converting it into kynurenine. Loss of TDO2 triggers a so far unknown compensatory mechanism, resulting in a vast increase of circulating kynurenine independent of hepatocyte intrinsic activity of the related IDO-enzymes. This study provides new insights into how inflammation reprograms metabolism of the liver and the kynurenine pathway during viral hepatitis.

## Introduction

The liver is a central metabolic organ and a key regulator of systemic metabolism [1,2]. Hepatocytes are the primary cell type of the liver and are responsible for the metabolic turnover of biomolecules, such as lipids, proteins and amino acids [3,4]. As a central organ, the liver is situated at the interface of metabolism, systemic inflammation and gut microbiota. Hence, it has to integrate immune responses to pathogens and immunogenic tolerance to commensals [4–6]. Hepatocytes are major coordinators of these processes, as they are not only metabolically highly active cells, but also important signaling platforms for innate and adaptive immunity [7–11].

Immune responses and metabolism are intimately linked processes. Particularly in the last decade, research in the field of immuno-metabolism has broadened our knowledge of how pathogens or cytokines initiate metabolic reprogramming of immune cells and thus license adequate immune responses. Many of these seminal discoveries were made using well-established and controlled *in vitro* model system [12,13]. Yet, to investigate immune-metabolic mechanisms in a disease context, it is inevitable to study systemic immunometabolism on the organismal level and account for cellular heterogeneity, a defining feature of tissues that enables them to adapt to changing environments and restore homeostasis [14,15,16].

Infectious diseases including chronic viral hepatitis are complex processes that involve crosstalk of multiple cell types, as well as a specific cytokine milieu [17]. Particularly the antiviral cytokine type I interferon (IFN-I) that signals through the ubiquitously expressed IFN-I receptor (IFNAR1) induces interferon stimulated genes (ISGs) that are critical to mount innate immune responses and limit viral replication [18]. Further, IFN-I contributes to metabolic reprogramming not only in immune cells [19–21], but also fibroblasts [20] or epithelial cells [21]. In hepatocytes, virus-induced IFN-I signaling regulates redox homeostasis and orchestrates wide-spread reprogramming of central metabolic pathways in hepatocytes, including lipid and amino acid metabolism [11,22]. During viral hepatitis, this specifically reduces circulating amino acids, resulting in repression of antiviral T cell responses and ameliorated T cell-mediated tissue damage [11]. Yet, the direct effects of IFN-I signaling on reprogramming of hepatocyte metabolism and its impact on antiviral immunity remain elusive.

Here, we employ unbiased transcriptome analyses to identify IFN-I-mediated metabolic reprogramming of hepatocytes and characterize them in disease-relevant model systems. To this end, we complement well-controlled primary cell culture models *in vitro* with the murine infection model of lymphocytic choriomeningitis virus clone13 (LCMV Cl13) and gene-targeted animals *in vivo* [23]. LCMV Cl13 establishes a chronic systemic infection and also infects hepatocytes, leading to T cell mediated hepatitis [24–27]. We identify IFN-I as sufficient and necessary to induce wide-spread metabolic reprogramming in hepatocytes and specific activation of the hepatic tryptophan-kynurenine pathway via *Tdo2*. The resulting infection-associated drop of the serum tryptophan to kynurenine ratio seems to be highly compensated during viral infection *in vivo*.

## Results

### Type I interferon modulates metabolic pathways in hepatocytes

**Fig 1:**
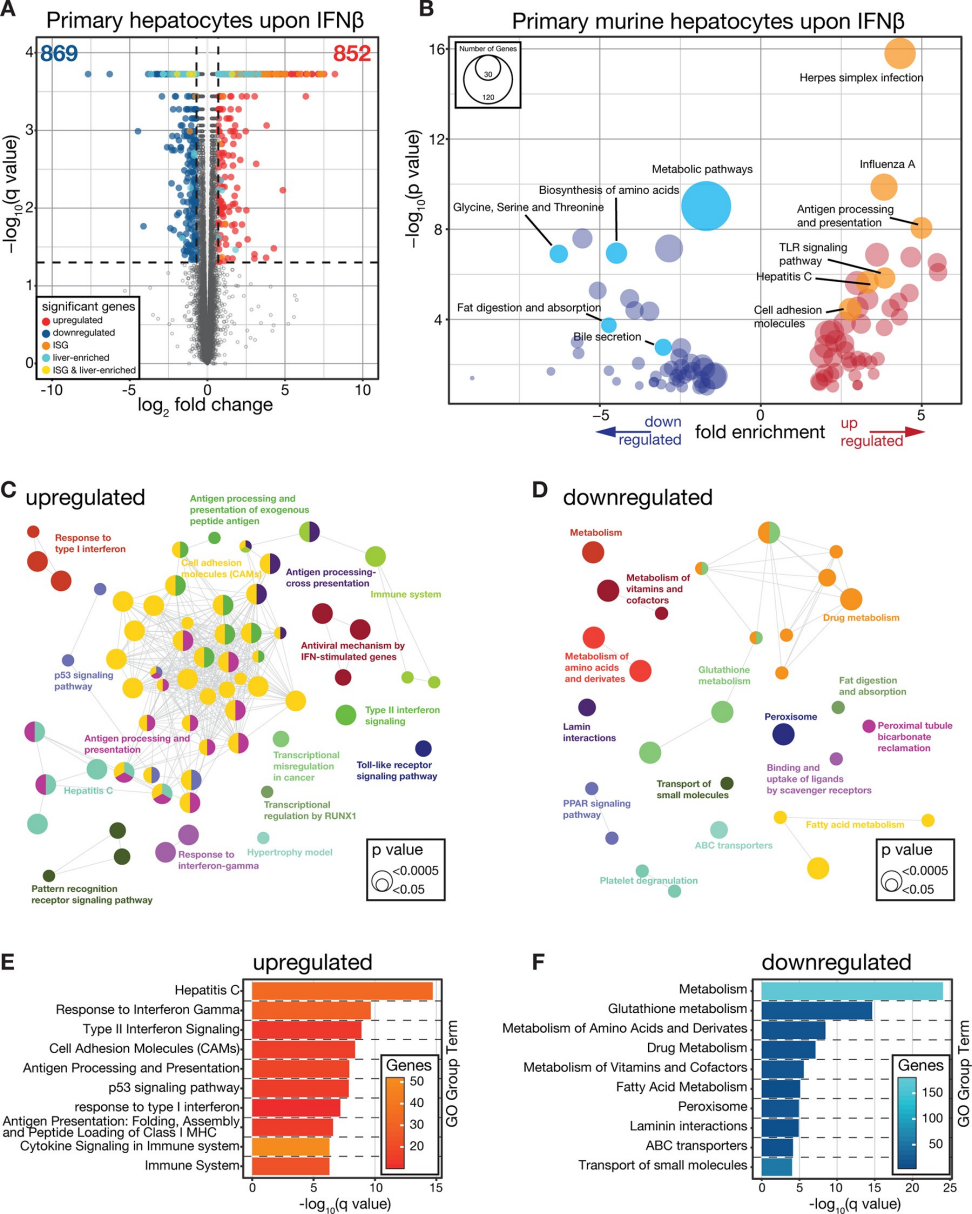
Type I interferon induces robust antiviral immune responses and metabolic reprogramming in primary hepatocytes. (A) Differential gene expression analyses of primary murine hepatocytes treated with IFNβ for 24 hours compared to untreated controls (n = 3). (B) KEGG pathway enrichment via DAVID database of significantly regulated transcripts (absolute log_2_ fold change > 0.7, adj. p value < 0.05). (C-D) GO term analyses of up- and downregulated transcripts based via ClueGO enrichment analyses. (E-F) Enriched GO group terms (ClueGO) of significantly regulated transcripts.

Type I interferon (IFN-I) signaling is critical for mounting innate antiviral immunity. We recently identified that virus-induced hepatocyte-intrinsic IFNAR1 is required for infection-associated metabolic reprogramming of hepatocytes [11]. This prompted us to investigate, whether IFN-I alone is sufficient to regulate metabolic pathways in hepatocytes. To this end, we isolated primary hepatocytes from C57Bl/6J (wild type) mice and treated them with IFNβ (1000 U/mL) for 24 hours and performed RNA sequencing analyses (RNA-seq). We identified 1,721 differentially regulated transcripts and found induced genes enriched for interferon stimulated genes (ISGs) and repressed genes enriched for liver-associated genes (Fig 1A and S1A Fig). An enrichment analyses based on Kyoto Encyclopedia of Genes and Genomes (KEGG) via the Database for Annotation, Visualization and Integrated Discovery (DAVID) [28] identified significantly up- and downregulated genes as being enriched for antiviral–and metabolic signaling pathways, respectively (Fig 1B). Similarly, a ClueGO [29] enrichment analyses particularly highlighted IFN-I-induced metabolic reprogramming of amino acid metabolism-associated pathways in hepatocytes (Fig 1C–1F). These data highlight that IFN-I alone is sufficient to induce wide-spread metabolic gene expression changes in hepatocytes.

### Type I interferon induces *Tdo2* and the tryptophan-kynurenine pathway in hepatocytes

**Fig 2:**
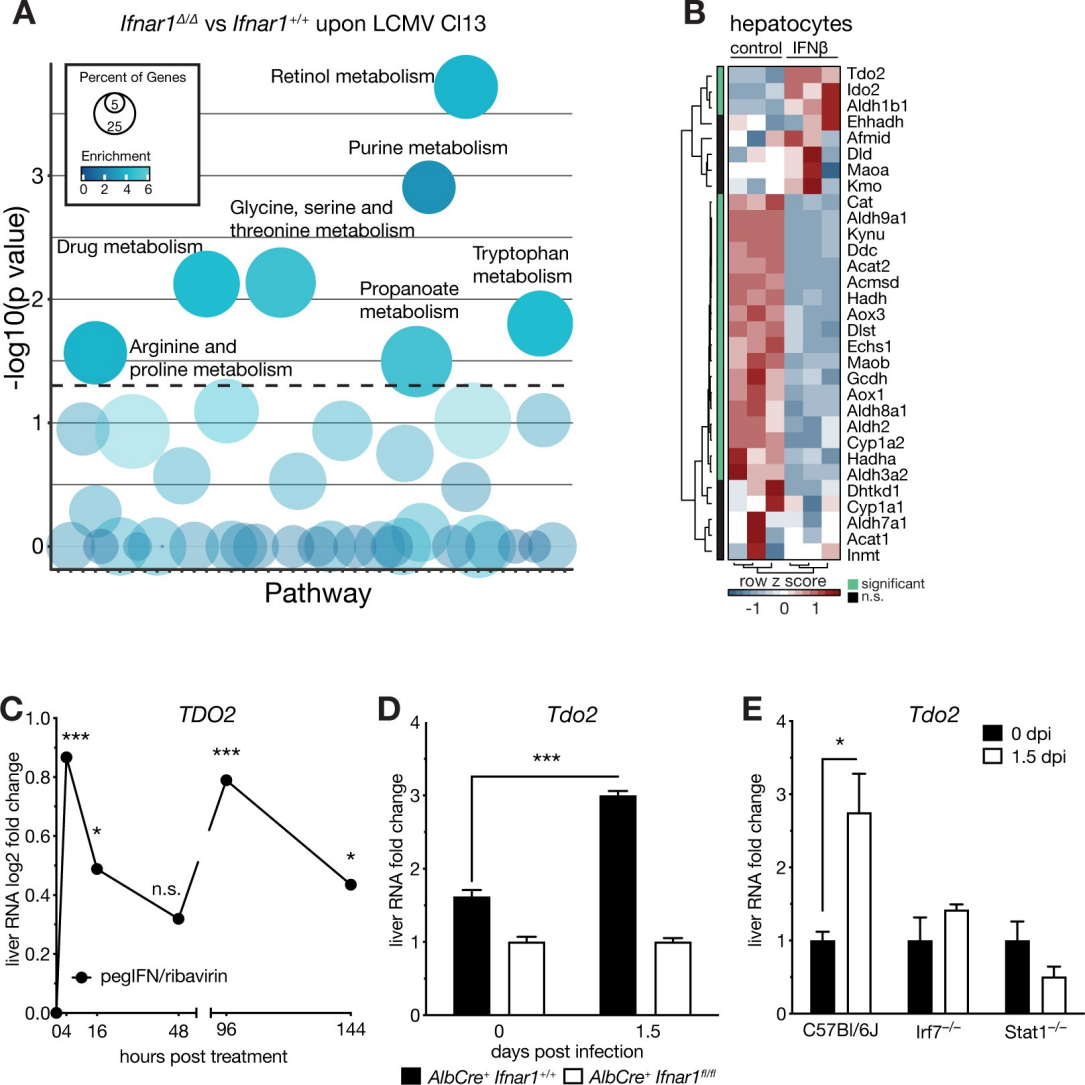
Type I interferon signaling induces Tdo2 and the tryptophan-kynurenine pathway in hepatocytes. (A) Metabolic pathway (KEGG database) enrichment analyses of differentially expressed transcripts in liver tissue of LCMV clone 13 infected (1.5dpi) versus naïve *Alb-Cre ERT2 Ifnar1*^*fl/fl*^ (*Ifnar1*^*Δ/Δ*^) and *Ifnar1*^*+/+*^ mice (n = 3). (B) Hierarchical clustering of expression levels of tryptophan metabolism associated genes (KEGG, k-means, Pearson’s Heatmap) in primary murine hepatocytes 24h after IFNβ treatment. (C) *Tdo2* transcript levels in liver tissue of hepatitis C virus infected patients upon treatment with pegylated type I interferon. (D) *Tdo2* expression levels in naïve and LCMV clone 13 infected (1.5dpi) *Ifnar1*^*Δ/Δ*^ and *Ifnar1*^*+/+*^ mice (n = 3) measured via RT-qPCR. (E) *Tdo2* expression levels in in naïve and LCMV clone 13 infected (1.5dpi) wild type (C57Bl/6J), *Irf7*^*–/–*^and *Stat1*^*–/–*^mice (n = 3). Symbols represent the arithmetic mean ±S.E.M. ns = not significant * P < 0.05 ** P < 0.01 *** P < 0.001 (Student’s t-test).

Next, we re-analyzed expression changes of metabolic genes in RNA-seq datasets from bulk liver tissue of LCMV Cl13 infected hepatocyte-specific IFNAR1 knock out (*ERT2-AlbCre*^*+*^ *Ifnar*^*fl/fl*^, knock out, *Ifnar1*^*Δ/Δ*^) and control (*ERT2-AlbCre*^*+*^ *Ifnar1*^*+/+*^, wild type) mice [11]. Apart from pathways related to retinol, purine or drug metabolism, IFN-I predominantly targeted metabolic pathways of amino acids, including arginine, proline, glycine, serine, threonine and tryptophan (Fig 2A). The liver is a central hub of amino acid metabolism and, amongst others, controls serum homeostasis of the essential amino acid tryptophan [2,3,30,31]. Strikingly, expression of *Tdo2*, *Ido2* and *Aldh1b1* was found to be significantly higher in IFNβ-treated primary hepatocytes, whereas the majority of tryptophan pathway-associated genes (KEGG) were repressed upon stimulation with IFNβ (Fig 2B). *Tdo2* encodes for the liver-specific, rate-limiting enzyme of the kynurenine pathway that oxidizes tryptophan to kynurenine. The closely related gene *Ido1* was not found to be expressed in hepatocytes, whereas its related gene *Ido2* was similarly induced (Fig 2B). Likewise, expression levels of *Tdo2* are elevated in liver tissue of hepatitis C virus (HCV) patients that received IFN-I (pegylated IFNα) treatment compared to untreated HCV-negative controls (Fig 2C) [32]. Contrary to our experimental data, *Ido2* was not found induced upon IFN-I treatment of HCV patients in this dataset (S2A Fig). In line with our *in vitro* results and the HCV data, infection of *Ifnar1*^*Δ/Δ*^ and *Ifnar1*^*+/+*^ control mice with LCMV Cl13 led to induction of *Tdo2* expression in wild type, but not in conditional IFNAR1 knock out mice (Fig 2D). Induction of *Tdo2* peaked at 1.5 days post infection (dpi), following IFN-I serum kinetics [22] (S2B Fig). To test whether *Tdo2* is induced via canonical IFNAR1 downstream signaling, we infected *Stat1*- and *Irf7*-deficient animals and found no upregulation of *Tdo2* at 1.5dpi (Fig 2E).

These data highlights that cell-intrinsic canonical IFN-I signaling is sufficient and necessary to induce the liver-specific tryptophan-degrading enzyme *Tdo2* in hepatocytes, which is in stark contrast to most other metabolic genes in the liver.

### Viral infection leads to sustained induction of the hepatic tryptophan-kynurenine pathway

**Fig 3:**
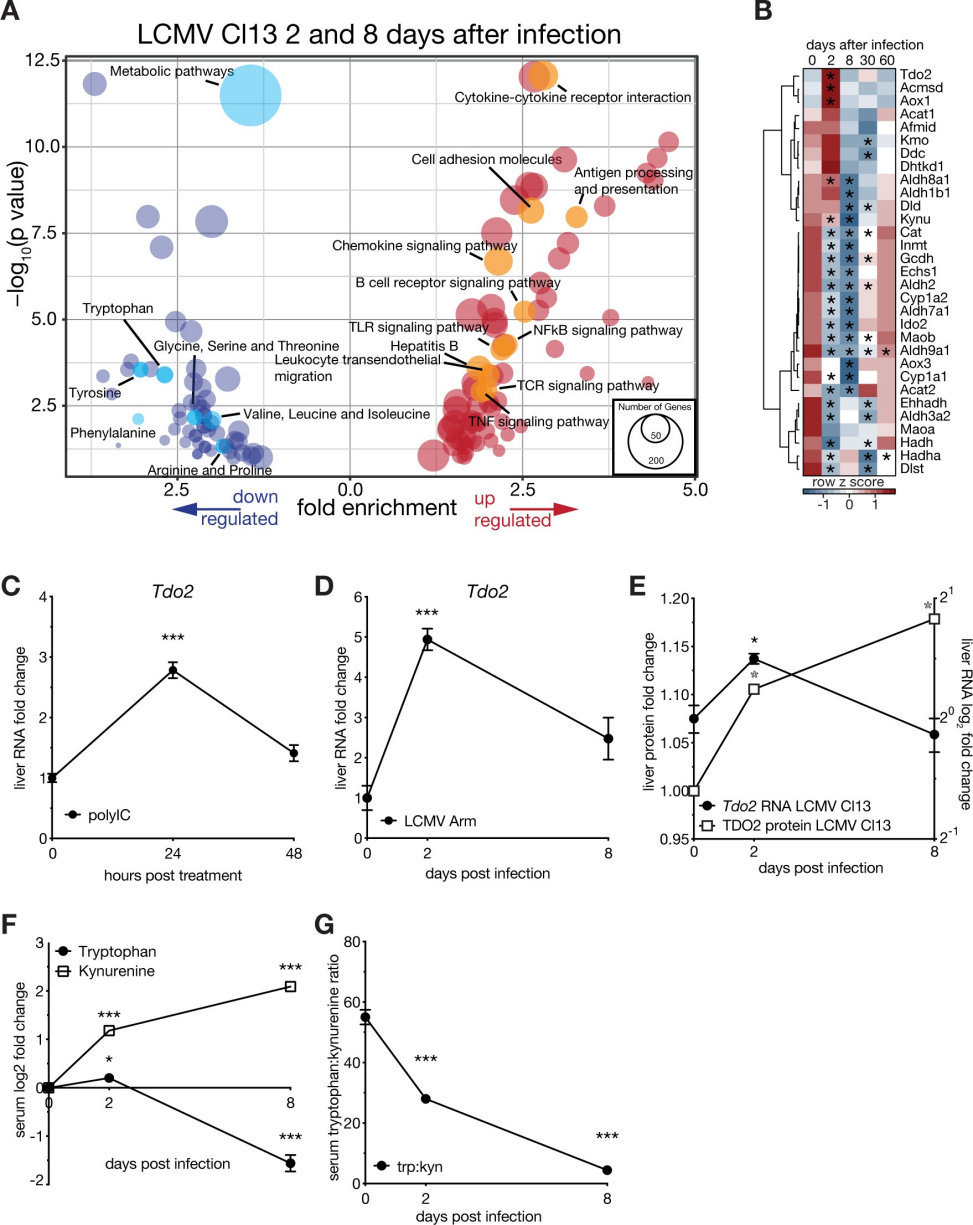
Viral infection leads to prolonged reprogramming of the hepatic tryptophan-kynurenine pathway and correlates with serum metabolite levels. (A) Metabolic pathway (KEGG database) enrichment analyses of differentially expressed transcripts in liver tissue of LCMV clone 13 infected (union of 2 and 8 days after infection) wild type (C57Bl/6J) mice. (B) Hierarchical clustering of expression levels of tryptophan metabolism associated genes (KEGG, k-means, Pearson’s Heatmap) in liver tissue of LCMV clone 13 infected animals at the indicated time points. (C) *Tdo2* transcript levels in liver tissue of polyI:C treated (24 and 48 hours) or (D) LCMV strain Armstrong 53b infected (2 and 8 days after infection) wild type (C57Bl/6J) animals measured via RT-qPCR (n = 3–4). (E) *Tdo2* transcript and protein levels in liver tissue of LCMV clone 13 infected (2 and 8 days after infection) wild type (C57Bl/6J) animals measured via RNA sequencing and quantitative proteomics (n = 3). (F) Tryptophan to kynurenine concentration and (G) ratio in the serum of LCMV Cl13 infected wild type mice at 0, 2 and 8dpi (n = 4). Symbols represent the arithmetic mean ±S.E.M. ns = not significant * P < 0.05 ** P < 0.01 *** P < 0.001 (Student’s t-test).

We recently published multi-omics datasets (GSE123688), describing transcriptomic and proteomic changes in the liver as well as serum metabolite changes during LCMV Cl13 infection [11]. We performed KEGG pathway enrichment analyses via the DAVID database on significantly regulated liver transcripts of 2 (~ innate phase) and 8 (~ acute phase) days after infection. Apart from an upregulation of immune-related pathways, we identified a drastic downregulation of metabolic pathways. Among those, most of the amino acid metabolic pathways were enriched (Fig 3A). Specifically, viral infection repressed most tryptophan metabolism-associated genes in the liver up to 8 days after infection, but specifically induced *Tdo2* (Fig 3B). Similar to *Tdo2*, the genes *Acmsd* and *Aox1* were significantly upregulated in liver tissue in the innate phase of infection (Fig 3B). This appeared to be independent of hepatocyte-intrinsic IFN-I signaling (Fig 2B). Thus, *Acmsd* and *Aox1* might be regulated by other non-epithelial cell types in the liver at this time point after infection. Downstream genes of *Tdo2*, including *Kmo*, *Kynu*, *Maoa* and *Maob* were either not found to be differentially expressed or downregulated by IFN-I signaling in hepatocytes or viral infection of the liver (Figs 2B and 3B). Mining a single cell RNA-seq (scRNA-seq) dataset (GSE123688) confirmed a significant infection-induced upregulation of *Tdo2* in hepatocytes and further confirmed that *Ido1* is not expressed whereas *Ido2* seems to be very lowly expressed in hepatocytes and not differentially regulated (S3A–S3E Fig). In line with our previous data, expression of the *Tdo2*-related gene *Ido1* and its protein IDO1 were not detected in liver tissue nor primary hepatocytes via RNA-seq (S3F–S3I Fig). Additionally, infection led to a repression of *Ido2* on the transcriptomic and proteomic level in the liver (S3F–S3J Fig).

These data suggest reprogramming of tryptophan metabolism towards kynurenine via *Tdo2* and virus-induced IFN-I. To corroborate this finding, we treated wild type (WT) mice with the viral PAMP analogue polyinosinic:polycytodylic acid (polyI:C) and found that this was sufficient to induce *Tdo2* expression (Fig 3C). Infection with the non-hepatotropic LCMV strain Armstrong 53b (LCMV Arm) induced *Tdo2* expression in the liver (Fig 3D). Upon LCMV Cl13 infection, expression of *Tdo2* peaked after 2 days and TDO2 protein levels remained significantly elevated up to 8 days after infection (Fig 3E). Accordingly, infection led to a decrease of tryptophan and increase of kynurenine levels in the serum (Fig 3F). The virus and IFN-I-mediated induction of TDO2 expression correlated with a drop of the systemic tryptophan to kynurenine ratio that was lowest 8 days after infection (Fig 3G).

### Viral infection induces compensatory mechanisms in *Tdo2*-deficient animals to maintain serum metabolite homeostasis

**Fig 4:**
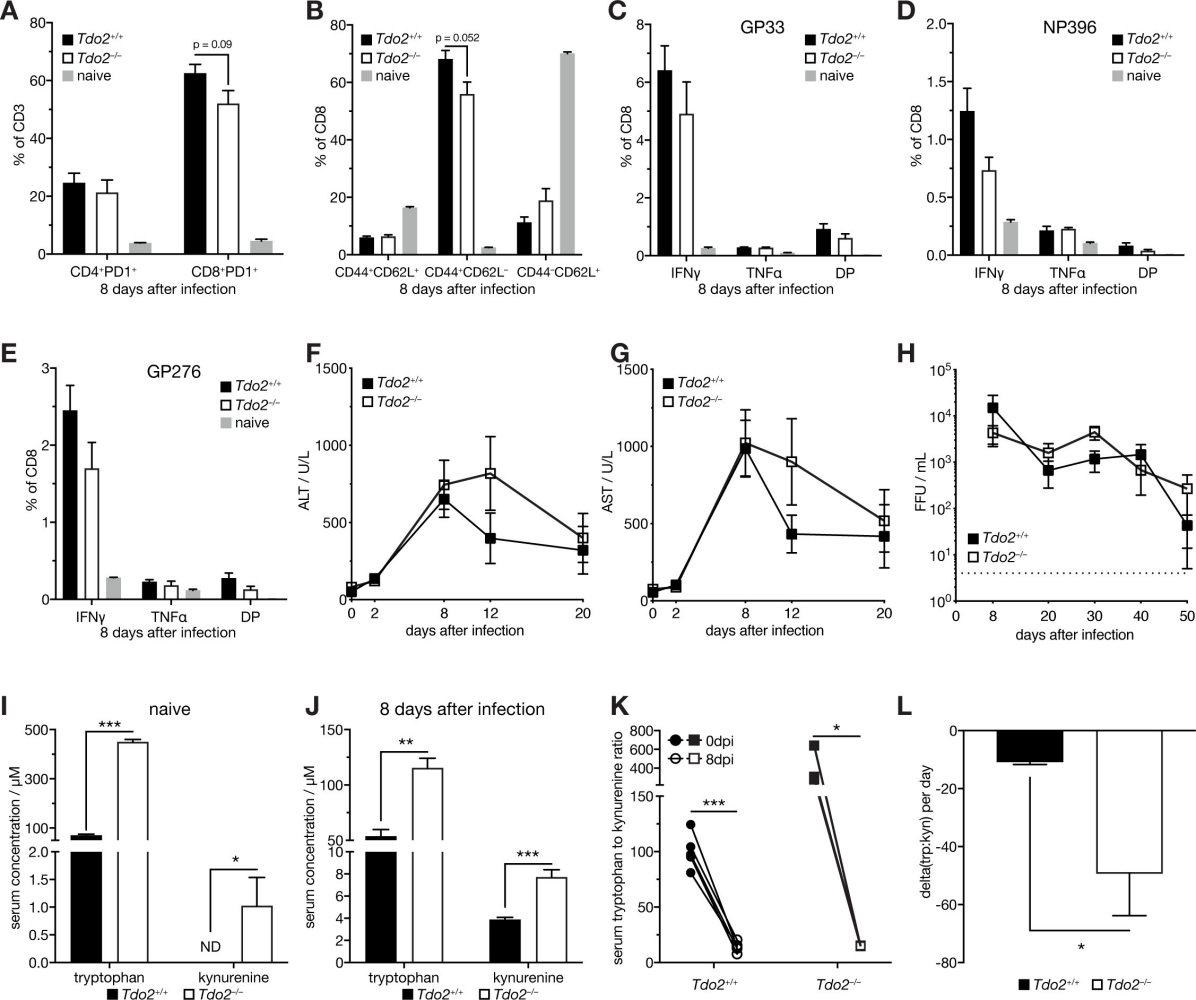
*Tdo2*-deficiency leads to mild changes in antiviral T cell responses in viral hepatitis and does not affect the serum tryptophan to kynurenine ratio upon infection. (A) Abundance of PD1 positive splenic CD4 and CD8 T cells in LCMV clone 13 infected (8 days after infection) *Tdo2*-deficient animals compared to littermate wild type animals (n = 3–5). (B) CD62L^+^CD44^+^ (memory), CD62L^–^CD44^+^ (effector) and CD62L^+^CD44^–^ (naïve) CD8 T cells (n = 3–5). (C-E) IFNγ and TNFα production of LCMV GP33-, NP396- and GP276-specific splenic CD8 T cells (n = 3–5). (F) Serum alanine aminotransferase (ALT), (G) aspartate aminotransferase (AST) levels and (H) viral load in blood in LCMV clone 13 infected *Tdo2*-deficient animals compared to littermate controls (n = 5). (I) Naive and (J) infected (8 days after infection) serum levels of tryptophan and kynurenine in *Tdo2*-deficient animals compared to littermate wild type animals (n = 3–5). (K) Serum tryptophan to kynurenine ratio and (L) slope of the drop of the serum tryptophan to kynurenine ratio in *Tdo2*-deficient animals compared to littermate wild type animals (n = 3–5). Symbols represent the arithmetic mean ±S.E.M. ns = not significant * P < 0.05 ** P < 0.01 *** P < 0.001 (Student’s t-test).

To test whether TDO2 affects pathophysiology in a model of viral hepatitis, we infected *Tdo2*-deficient mice [30] and littermate wild type controls (*Tdo2*^*–/–*^and WT) with 2x10^6^ focus forming units (FFU) of LCMV Cl13. *Tdo2*^*–/–*^and WT mice did not display any differences in viremia in blood, liver, spleen or kidney 2 days after infection (S4A Fig). RT-qPCR analyses of liver tissue showed no difference in expression of the classical IFN-I stimulated gene (ISG) *Ifit1* (S4B Fig), in line with comparable serum levels of IFNα in *Tdo2*^*–/–*^and WT mice at 2dpi (S4C Fig). TDO2 protein levels (Fig 3E) and serum tryptophan to kynurenine ratio were regulated up to 8 days after infection (Fig 3F), coinciding with the peak of antiviral T cell responses in the LCMV infection model [33]. We did not observe differences in overall splenic CD4^+^ and CD8^+^ T cell abundance and expression of the early activation marker PD1 in naive *Tdo2*^*–/–*^compared to WT animals (S4D Fig). Yet, T cells from naive *Tdo2*^*–/–*^mice expressed slightly reduced levels of the activation marker CD44 and increased levels of the naive T cell marker CD62L (S4E and S4F Fig). T cell responses at 8dpi were widely comparable and we did not observe significant differences in splenic CD4^+^ or CD8^+^ T cell abundances and proportion of virus-specific CD8^+^ T (S4G and S4H Fig). However, splenic CD8^+^ T cells of *Tdo2*-deficient animals expressed slightly reduced levels of PD1 (Fig 4A). Similarly, a tendency towards reduced CD8^+^ effector memory (CD44^+^ CD62L^–^) and increased naive (CD44^–^ CD62L^+^), was observed in *Tdo2*^*–/–*^animals, whereas central memory (CD44^+^ CD62L^+^) T cells remained unchanged (Fig 4B). Further, LCMV-specific CD8^+^ T cells from *Tdo2*^*–/–*^animals recognizing the immune-dominant epitopes GP_33-41_, NP_396-404_ and GP_276-284_ showed a minute reduction in the production of the effector cytokines IFNγ and TNFα at 8dpi (Fig 4C–4E), which was maintained up to 50dpi (S4I–S4K Fig). Serum levels of the liver tissue damage markers alanine aminotransferase (ALT) and aspartate aminotransferase (AST), as well as serum levels of albumin did not significantly differ between *Tdo2*^*–/–*^and WT littermates (Fig 4F and 4G and S4L Fig). Together, these data suggest that loss of TDO2 does not impact overall antiviral immunity or pathophysiology of virus-induced hepatitis. Conversely, we did not observe significant differences in viral load in the blood (Fig 4H), nor in viremia and viral RNA loads in liver, spleen or kidney at 8dpi or 50dpi (S4M–S4O Fig), suggesting comparable viral control in *Tdo2*-deficient and WT animals. Since *Tdo2* is an important regulator of circulating tryptophan [31], which is important for T cell activation and proliferation [34,35], these findings were somewhat unexpected. We performed additional serum metabolite analyses of naive *Tdo2*^*–/–*^and WT littermate controls and could confirm strongly elevated tryptophan and, counterintuitively, slightly increased kynurenine levels (Fig 4I). A similar observation has been previously made by an independently generated *Tdo2*-deficient mouse [31]. Serum concentrations of tryptophan and, surprisingly, kynurenine were significantly increased in infected *Tdo2*^*–/–*^animals compared to WT animals (Fig 4J). These data show that even in the absence of TDO2, infection leads to reduction of the serum tryptophan to kynurenine ratio (Fig 4K), which appeared to be even more pronounced in *Tdo2*^*–/–*^animals (Fig 4L). Consequently, there is no significant difference in the serum tryptophan to kynurenine ratio between *Tdo2*-deficient and WT animals at 8 days after infection (S4P Fig).

### The IDO-axis in hepatocytes is not involved in compensating loss of TDO2

**Fig 5:**
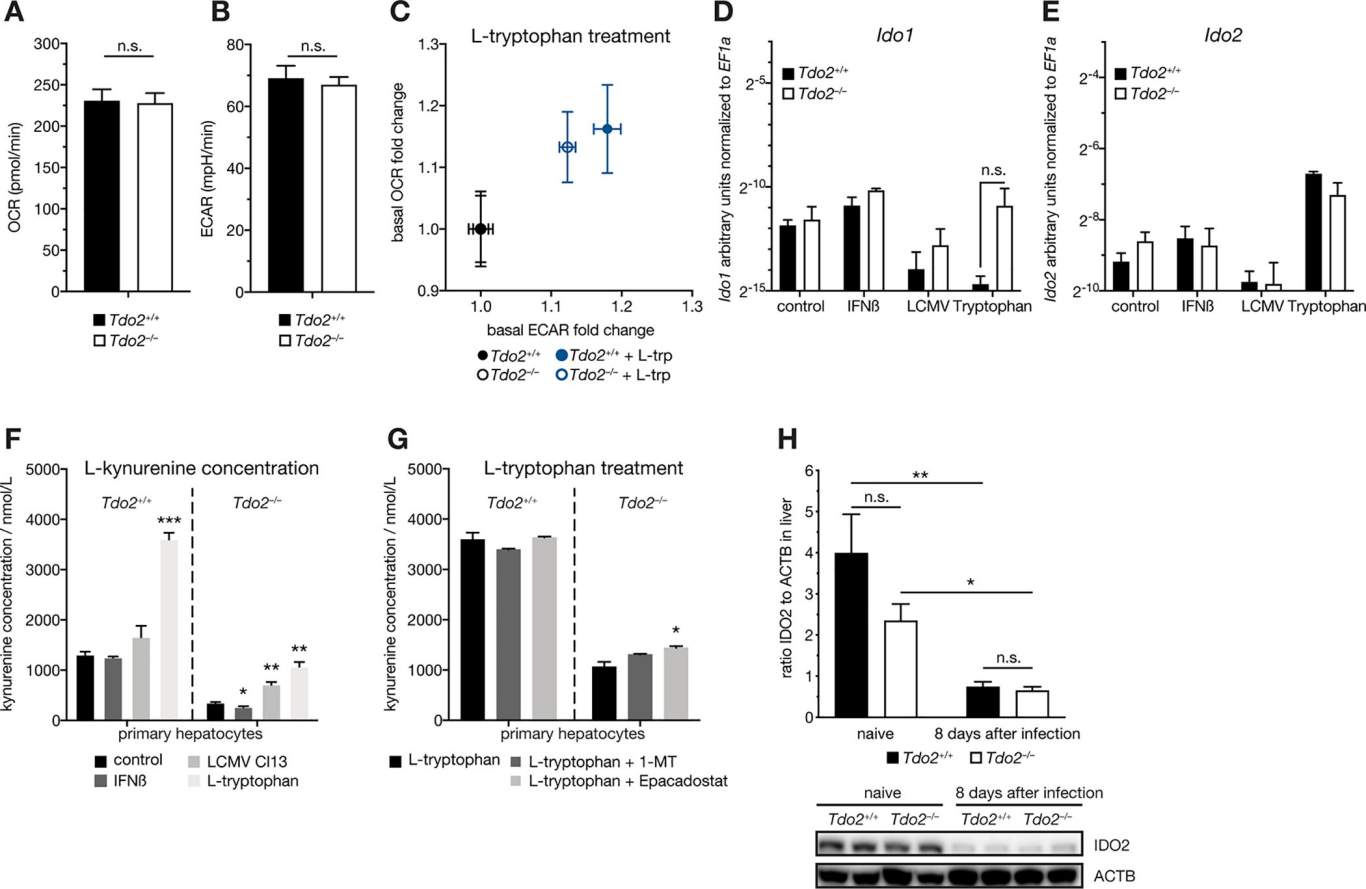
The IDO mediated tryptophan degradation in hepatocytes is not involved in compensating loss of TDO2. (A) Oxygen consumption rate (OCR) and (B) extracellular acidification rate (ECAR) of *Tdo2*-deficient primary hepatocytes compared to wild type littermate controls (n = 11–12). (C) OCR and ECAR upon L-tryptophan (1 mM) stimulation (n = 11–12). (D) *Ido1* and (E) *Ido2* transcript levels in *Tdo2*-deficient primary hepatocytes and wild type littermate controls and upon IFNβ (1000 U/mL), L-tryptophan (1 mM) or LCMV clone 13 infection (MOI 3) (n = 3). L-kynurenine concentration in supernatants of *Tdo2*-deficient and wild type primary hepatocytes upon (F) IFNβ (1000 U/mL) treatment or LCMV clone 13 infection (MOI 3) and (G) with or without the IDO-inhibitor 1-methyl-L-tryptophan (1-MT, 1 mM) or the IDO1 inhibitor Epacadostat (500 nM) (n = 3). (H) IDO2 protein quantification (n = 4) and representative western blot (n = 2) from liver tissue of LCMV clone 13 infected (8days after infection) *Tdo2*-deficient and wild type littermate controls. Symbols represent the arithmetic mean ±S.E.M. ns = not significant * P < 0.05 ** P < 0.01 *** P < 0.001 (Student’s t-test).

Elevated kynurenine serum levels in *Tdo2*-deficient animals may be caused by multiple and not mutually exclusive factors including altered metabolite transport, microbiome and tryptophan and/or kynurenine utilization. Next to TDO2, IDO1 and, to a lesser extent, IDO2 can convert tryptophan to kynurenine [36]. We thus considered the possibility that loss of the liver-specific enzyme TDO2 might initiate a hepatocyte-intrinsic compensatory mechanism via the IDO-axis. To this end, we isolated primary wild type hepatocytes and infected them with LCMV Cl13 (MOI 3) or stimulated them with IFNβ (1000 U/mL) or tryptophan (1 mM) for 24 hours. All conditions resulted in induced gene expression of *Tdo2* (S5A Fig). Next, we assessed the metabolic state in primary *Tdo2*^*–/–*^and WT hepatocytes by determining oxygen consumption (OCR) and extracellular acidification rates (ECAR) (Fig 5A and 5B). Both genotypes displayed similar metabolic activities. Intriguingly, this was also the case when stimulating *Tdo2*^*–/–*^and WT hepatocytes with tryptophan, suggesting increased metabolic activity (Fig 5C). Comparison of *Ido1* and *Ido2* gene expression levels upon LCMV Cl13 infection, IFNβ or tryptophan treatment revealed no genotype-specific differences (Fig 5D and 5E). To assess tryptophan degradation and kynurenine secretion, we quantified kynurenine concentrations in cell culture supernatants of the respective conditions. Supernatants of *Tdo2*^*–/–*^hepatocytes contained significantly less kynurenine compared to WT hepatocytes (Fig 5F). Addition of 1 mM tryptophan led to a significant elevation of extracellular kynurenine levels in *Tdo2*^*–/–*^hepatocytes (Fig 5F). To investigate, whether this increase was independent of IDO1 and IDO2, we treated cells with the IDO1/2 inhibitor 1-methyl-L-tryptophan (1-MT, 1 mM) or the IDO1-specific inhibitor Epacadostat (INCB024360, 500 nM) [37]. Neither inhibitor reduced kynurenine levels in unstimulated or tryptophan stimulated hepatocytes compared to the respective controls (S5D Fig and Fig 5G). On an organ level, LCMV infection did not alter expression levels of *Ido1* and *Ido2* in liver, spleen or kidney tissue of *Tdo2*^*–/–*^and WT animals (S5B and S5C Fig). In line with our presented transcriptomic and proteomic data, IDO1 is not expressed in naive and LCMV-infected liver tissue of *Tdo2*^*–/–*^or WT mice (S5E Fig), whereas IDO2 protein levels are reduced 8 dpi in both genotypes (Fig 5H). Together, these data suggest that the observed systemic increase of kynurenine during homeostasis and upon viral infection of *Tdo2*-deficient animals is unlikely to be due to altered hepatocyte-intrinsic activity of IDO1 or IDO2.

## Discussion

Extracellular amino acids critically regulate immune responses [35,38–40]. This can have i) beneficial effects for the host in order to reduce collateral damage [41] or ii) be detrimental when immunity fails to control pathogens or tumors [42]. The liver plays a central role in amino acid metabolism and controls serum levels of several metabolites during homeostasis and viral infection [1,2,11]. This includes the essential amino acid tryptophan, where serum availability is associated with neurologic dysfunctions and inflammation [31,43]. The hepatic kynurenine pathway and in particular the tissue-specific enzyme TDO2 are central in degrading excess tryptophan and in maintaining tryptophan homeostasis in the circulation [31,44]. Low tryptophan levels are thought to be sensed mainly by GCN2 via uncharged tRNAs, but recent studies suggested a GCN2-independent mechanism, hinting towards alternative tryptophan sensors [34,45,46]. The downstream products of the kynurenine pathway and particular kynurenine derivates have well-established immune-suppressive properties that are mainly sensed by the intracellular aryl hydrocarbonate receptor (AhR) [47].

In other tissues, the conversion of tryptophan to kynurenine can be catalyzed by IDO1 or to a lesser extent by the closely related protein IDO2 [36]. Generally, serum tryptophan to kynurenine ratio is a parameter for activity of the kynurenine pathway at the organismal level [48]. During homeostasis, the serum tryptophan to kynurenine ratio is primarily affected by TDO2 and not by IDO1 or IDO2 activity [30,31,49]. Reduced serum tryptophan to kynurenine ratios are observed during multiple human viral infections, including human immunodeficiency virus (HIV) [50], hepatitis C virus (HCV) [51], dengue virus (DENV) [52] and the pandemic severe acute respiratory syndrome coronavirus 2 (SARS-CoV-2) [53]. The infection-associated drop of the serum tryptophan to kynurenine ratio might not be solely affected by altered TDO2 or IDO activities. Indeed, our *ex vivo* data generated in primary *Tdo2*-deficient hepatocytes combined with IDO inhibitors hint towards the existence of additional tryptophan oxidizing enzymes.

On a molecular level, IDO enzymes are structurally distinct from TDO2 and display a broader substrate-range, whereas TDO2 exclusively binds L-tryptophan [36]. IDO2 shows low catalytic activity and substrate specificity and is not thought to contribute to systemic tryptophan metabolism [36,44,49,54]. IDO1 is the only tryptophan-degrading enzyme with well-established immune-regulatory functions and represses T cell function via tryptophan-derived kynurenine derivates [55,56]. IDO1 is induced by IFNγ and mainly expressed by dendritic cells [55], thus generating a locally restricted regulatory circuit that is barely present in the liver [44]. We could not detect expression of *Ido1*, whereas *Ido2* appeared to be expressed in liver tissue or hepatocytes. Yet, *Ido2* was inconsistently regulated in our *in vitro* and *in vivo* model systems.

As a metabolic and immunological organ alike, the liver seems to be predestined to act as a central regulator of systemic amino acid metabolism during inflammation. IFN-I is an important antiviral cytokine and important for pathogen control during viral hepatitis [17,57]. IFN-I can also initiate metabolic reprogramming in immune cells and tissue-resident cells [11,19–21]. Virus-induced IFN-I triggers an endogenous regulatory circuit in hepatocytes to ameliorate tissue damage in viral hepatitis via increased local degradation and systemic repression of amino acids and accumulation of downstream metabolites [11].

Here, we report that treatment of primary murine hepatocytes with IFN-I *ex vivo* was sufficient to trigger wide-spread reprogramming of metabolic pathways. Strikingly, our complementary experimental models and integrated patient data from HCV infection suggest that IFN-I is sufficient and necessary to induce the tryptophan-kynurenine pathway via the liver-specific gene *Tdo2* in hepatocytes. Previous studies highlighted a potential beneficial role of *Tdo2* in endotoxemia [58], while certain tumors upregulate *Tdo2* to repress tumor-specific T cell responses [59–62]. This suggests that transcriptional regulation of *Tdo2* may be an immune-regulatory mechanism.

Similar to classic ISGs, IFN-I-mediated induction of *Tdo2* critically depended on the presence of the surface receptor IFNAR1 and the canonical downstream signaling via the adaptor molecule STAT1 and the IFN-I signaling master regulator IRF7 [57,63,64]. *Tdo2*-deficient animals did not display altered IFN-I responses in our viral infection model, which would argue against a direct regulatory feedback loop between IFN-I signaling and tryptophan-derived metabolites that was previously proposed in other model systems [65–68].

*Tdo2*-deficient animals display strongly elevated serum levels of tryptophan, but also elevated serum levels of kynurenine [30,31]. Upon viral infection, *Tdo2*-deficient mice retained significantly elevated serum tryptophan levels, which was accompanied by an unexpected drastic increase of serum kynurenine. These excess levels of kynurenine might cancel a supposedly positive effect of elevated tryptophan serum levels in *Tdo2*-deficient animals. Our data shows that *Tdo2*-deficiency is highly compensated during viral infection, resulting in maintenance of the infection-associated reduction of the systemic tryptophan to kynurenine ratio. *Tdo2*-deficiency did not result in altered expression or activity of IDO1 nor IDO2 in hepatocytes or whole liver tissue. Likewise, *Ido1* and *Ido2* are not differentially regulated in spleen or kidney tissue of naive or infected *Tdo2*-deficient animals. Thus, the observed compensation of *Tdo2*-deficiency with respect to the serum tryptophan to kynurenine ratio upon viral infection might be independent of the IDO-axis. Yet, since we found increased levels of IDO1 in LCMV-infected animals, extrahepatic compensatory mechanisms seem plausible. Other possible mechanisms may include i) altered uptake, utilization or excretion of tryptophan or kynurenine, ii) a yet unknown tryptophan degrading pathway that is repressed in the presence of TDO2 and/or iii) altered binding of tryptophan or kynurenine to serum proteins like albumin [48]. However, the latter seems unlikely, due to comparable serum albumin levels in uninfected and infected *Tdo2*^-/-^ and WT animals. The existence of compensatory mechanisms for the loss of *Tdo2* suggests a critical role for circulating tryptophan and kynurenine during infectious diseases and beyond. Conversely, the role of *Tdo2* in homeostasis and pathophysiology may benefit from experimental models with altered expression levels of *Tdo2* rather than loss of function models. The recent identification of IL4I1 as a tryptophan catabolizing enzyme further highlights the need for future investigations to unveil the full metabolic network regulating tryptophan homeostasis [69].

Together, our data conclusively show that canonical virus-induced IFN-I signaling initiates expression of *Tdo2* in hepatocytes. Moreover, we identified the hepatic kynurenine pathway as central metabolic node that is tightly regulated by so far unknown compensatory mechanisms in the absence of *Tdo2*.

## Materials and methods

### Contact for reagent and resource sharing

Inquires for additional information or requests for reagents should be directed and will be fulfilled by the lead contact Andreas Bergthaler (abergthaler@cemm.oeaw.ac.at).

### Mice

C57Bl/6J wild type mice were originally obtained from The Jackson Laboratory, *Ifnar1*^*fl/fl*^ [70], *Cre-Alb ERT2* [71], *Stat1*^*–/–*^[72], *Irf7*^*–/–*^[63], *Tdo2*^*–/–*^reporter [30] mice were bred and maintained on a C57Bl/6J background under specific pathogen-free (SPF) conditions at the Institute for Molecular Biotechnology (IMBA) of the Austrian Academy of Sciences, Vienna, Austria.

### Ethics statement

Animal experiments were performed in individually ventilated cages (IVC) at the Department for Biomedical Research of the Medical University of Vienna according to the respective animal experiment licenses (BMWFW-66.009/0199-WF/V/3v/2015 and BMWFW-66.009/0361-WF/V/3b/2017), approved by the institutional ethics committees and guidelines. Male and female mice were used interchangeably between experiments and no strong sex-specific differences were observed.

### Viruses and infection

Lymphocytic choriomeningitis virus (LCMV) stocks for the strains clone 13 and Armstrong 53b were grown on BHK-21 cells and titers were determined via a modified focus forming assay (FFA) using Vero cells [22]. Mice were intravenously infected with 2x10^6^ focus forming units (FFU) of LCMV and euthanized at the indicated time points. Tissue samples were snap frozen in liquid nitrogen and stored at -80°C for subsequent analyses.

### Conditional *Ifnar1* ablation on hepatocytes

Tamoxifen (T5658, Sigma) was dissolved in 10% ethanol (v/v) containing sunflower oil (S5007, Sigma, sterile) and stored at -20°C. for a maximum of two weeks Hepatocyte specific *Ifnar1* ablation was induced by administered 50 mg/kg tamoxifen intraperitoneally for 5 consecutive days (Metzger et al., 2005) to *Cre-Alb ERT2 Ifnar1*^*fl/fl*^ and *Cre-Alb ERT2 Ifnar1*^*+/+*^ control mice. All experiments were started the day after the last administration of tamoxifen.

### polyI:C treatment

Mice intraperitonally received 4 mg/kg polyinosinic-polycytidylic acid diluted in PBS (polyI:C, #tlrl-pic, invivogen). Control mice receive PBS and mice were harvested and tissue samples were analyzed at the indicated time points post treatment.

### Liver expression data of pegIFN-I treated hepatitis C patients

This gene expression dataset was previously published and analyzed [32]. In brief, a total of 25 patients participated in this study, of which 6 patients were hepatitis C virus (HCV) negative and thus liver tissue expression data of these samples served as naive controls. The 19 HCV positive patients were receiving ribavirin and pegylated IFN-I treatment according to HCV genotype body weight and standard treatment recommendation. These patients agreed to be biopsied after receiving the first treatment dose of pegylated IFN-I (1.5 μg/kg body weight of pegIFN-α-2b (Essex Chemie) or 180 μg of pegIFN-α-2a (Roche)), at 4 hours (5 patients), 16 hours (3 patients), 48 hours (3 patients), 96 hours (3 patients) and 144 hours (5 patients).

### Quantitative proteome analyses of liver tissue

This method and analyzes of the generated dataset was previously published [11]. In short, protein changes in LCMV-infected murine liver tissue was carried out via tandem mass tag (TMT) mass spectrometry. Liver tissues sampled from day 2 and day 8 after infection together with uninfected controls were analyzed within the same TMT 6-plex run (biological duplicates). To this, liver tissue samples were lyzed, processed using an adapted filter-aided sample preparation (FASP) method. The tissue lysates were reduced, buffer exchanged and digested with porcine trypsin (Promega). Samples were separately derivatized with TMT 6-plex reagents (Thermo Fisher Scientific) according to the instructions provided by the manufacturer. The TMT-labeled tryptic digests were pooled and concentrated by solid phase extraction (SPE) and separated on an Agilent 1200 series HPLC (Agilent Biotechnologies). Seventy-two time-based fractions were collected and after removal of organic solvent and reconstitution, the fractions where analyzed on an LCMS system, consisting of an Agilent 1200 nano-HPLC system (Agilent Biotechnologies) coupled to a hybrid LTQ Orbitrap Velos mass spectrometer (ThermoFisher Scientific) and data was acquired via Xcalibur software (version 2.1).

### Kynurenine measurements

Cell culture supernatants were collected and 300 μL were dried using a MiniVap (Porvair Sciences) instrument and resuspended in 11 μL distilled water and used for further analyses. L-Kynurenine concentrations were determined using a commercial L-Kynurenine ELISA kit (BA-E-2200, ImmunoSmol) according to the manufacturer’s instructions. Sample concentrations were calculated according to the standard curve.

### Western blot

Tissue samples (approx. 100mg) were lyzed in 200μL RIPA buffer (Tris-HCl pH 8.0, 150 mM NaCl, 1% NP-40, 0.5% sodium deoxycholate, 0.1% SDS) containing Halt Protease and Phosphatase inhibitor cocktail (Thermo Fisher Scientific) using a TissueLyzer II (Qiagen). After centrifugation for 15 minutes at 15,000 rpm at 4°C and 100μL supernatant was transferred into a new tube, and protein concentrations were determined using the Coomassie Protein Assay kit (Thermo Fisher Scientific) according to manufacturers instructions. Protein concentrations were adjusted to 2 mg/mL with PBS.

For cell pellets, 25μL RIPA buffer containing Halt Protease and Phosphatase inhibitor cocktail (Thermo Fisher Scientific) was used. Cells were lyzed by vortexing for 20 seconds. Afterwards, samples were centrifugation for 15 minutes at 15,000 rpm at 4°C and supernatants were transferred into a new tube.

Laemmli buffer (4x stock containing 8% SDS, 40% glycerol, 0.008% bromphenol blue, 0.25M Tris-Cl pH 6.8, 20% 2-mercaptoethanol) was added and previous to loading, samples were incubated for 5 min at 95°C. Proteins were analyzed via SDS-PAGE using Bolt 4–12% Bis-Tris Protein Gels (Thermo Fisher Scientific). Proteins were blotted (wet transfer) on Westran Clear signal PVDF membranes (Sigma Aldrich). In total, 20μg total protein or lysate of 100,000 cells were loaded per lane and the following antibodies were used to visualize target proteins: anti-IDO1 (Cell Signaling Technology #68572), anti-IDO2 (Sigma #SAB3701447), anti-ACTB (Sigma #A2066), HRP-coupled anti-mouse-IgG (Dako #P0260) and HRP-coupled anti-rabbit-IgG (Dako, #P0448). PageRulerTM Prestained Protein Ladder (Thermo Scientific) was used for protein size determination. HRP signal were detected with Pierce ECL Western blotting substrate (Thermo Fisher Scientific) or Amersham ECL select Western blotting detection reagent (GE Healthcare Life Sciences). Imaging was done on a ChemiDoc Imaging System (Bio-Rad) and images were analyzed with Image Lab software (Bio-Rad).

### Isolation and culture of primary murine hepatocytes

Primary hepatocyte isolation was performed as previously described [11]. In brief, mice were anesthetized, the liver was cannulated, and hepatocytes were isolated via a two-step perfusion protocol using HBSS (Gibco) supplemented with 0.5 mM EGTA (Sigma) and L15 medium (Gibco) containing 40 mg/mL Liberase (Roche).

Cell culture plates were coated with rat tail collagen (Corning, 50mg/L in MilliQ water and 0.2N acetic acid) for 2h at 37°C. Hepatocytes were isolated from 3 individual mice, pooled and plated in 6 wells at a density of 600,000 cells per well in a volume of 2 mL William’s E medium (Gibco supplemented with 10% FCS (PAA) and 1% Penicillin-Streptomycin-Glutamine (PSQ, Thermo Fisher Scientific). After 4 hours, medium was exchanged for 1.5 mL William’s E medium (Gibco) supplemented with 0.5% FCS (PAA) and 1% PSQ (Thermo Fisher Scientific) containing the respective stimuli (1000 U/mL IFN-β, PBL; 3 MOI LCMV Cl13; 1 mM L-Tryptophan, Sigma). The IDO inhibitors 1-methyl-L-tryptophan (1-MT, #447439 Sigma) and Epacadostat were dissolved in PBS and DMSO and, according to literature [37], used at final concentrations of 1 mM and 500 nM, respectively. After 24 hours, the medium was collected, cells were washed with 1 mL cold PBS and harvested in 1 mL cold PBS via scraping. Of this, 200μL were separated, cells pelleted and resuspended in 1 mL QIAzol (Qiagen) and RNA was isolated.

### Metabolic flux measurements

Oxygen consumption rate (OCR) and extracellular acidification rate (ECAR) were measured on a Seahorse XFe96 platform (Agilent) using the Seahorse XF Cell Mito Stress test kit (Agilent) according to manufacturer’s instructions. In brief, 25.000 primary hepatocytes were plated per well and immediately treated for 4 hours with the respective stimuli, before proceeding with metabolic flux measurements. Prior to measurement, media was changed to XF Base Medium (Agilent) containing glucose (10 mM), sodium pyruvate (1 mM) and L-glutamine (2 mM) and cells were incubated for 1h. The compounds oligomycin (2 μM), Carbonyl cyanide-p-trifluoromethoxyphenylhydrazone (FCCP, 0.25 μM) and Rotenone/Antimycin A (500 nM) were previously titrated [11]. Raw data were analyzed using Wave Desktop Software (Agilent, version 2.0) and exported and graphed in GraphPad Prism (GraphPad Sorftware, version 7.0a).

### Blood chemistry

Mouse serum was pre-diluted 1:8 in PBS and levels of alanine aminotransferase (ALT) and aspartate aminotransferase (AST) were spectrophotometrically analyzed using a Cobas C311 Analyzer (Roche).

### Serum IFN-α determination

Pre-diluted serum samples (1:10 to 1:20 in PBS) were determined via enzyme-linked immunosorbent assay (ELISA) as previously described [22].

### RNA isolation and real-time PCR

Cell culture samples were directly harvested in QIAzol (Qiagen). Tissue samples were harvested in QIAzol (Qiagen) and homogenized using a TissueLyzer II (Qiagen). Total RNA was extracted according to the manufacturer’s instructions (79306, Qiagen). The First Strand cDNA Synthesis Kit (K1612, Thermo Fisher Scientific) was used to reverse transcribe the isolated RNA into cDNA. For gene expression analyses via real-time polymerase chain reaction (PCR), Taqman Fast Universal PCR Mastermix (4352042, Thermo Fisher Scientific) and Taqman Gene Expression Assays (Thermo Fisher Scientific) for *Tdo2* (Mm00451269_m1), *Ido1* (Mm00524210_m1), *Ido2* (Mm004925901_m1) and *Ifit1* (Mm00515153_m1) were used. Expression levels of LCMV NP (5’-CAAGTATTCACACGGCATGGA-3’, 5’-TGGGAGAGCACCTATAACTGATA-3’ and 5’-[6FAM]TGATCTCTTCAATGCACAGCCTGGGC[BHQ1]-3’) and *Ef1α* (5’-GCAAAAACGACCCACCAATG-3’, 5’-GGCCTTGGTTCAGGATA-3’, and 5’-[6FAM]CACCTGAGCAGTGAAGCCAG[TAM]-3’) were measured by corresponding probe and primer sets as described previously [22].

### Flow cytometry

Whole spleens were dissociated into a single cell suspension using a 40 μm cell strainer (Falcon) and resuspended in 10 mL of PBS per spleen (Gibco). An aliquot of the obtained cell suspensions was used for counting and calculating total number of cells per spleen. Per sample, 200 μL (approx. 2x10^6^ cells) were plated in a round bottom non tissue culture treated 96 well plate. Plates were spun and supernatants were discarded.

For tetramer staining, cells were resuspended in 25 μL PBS containing GP33 (1:500) and NP396 (1:250) tetramers (NIH Tetramer Core Facility) and incubated at 37°C for 15 min. To block Fc receptors, 25 μL PBS containing anti-CD16/32 (Biolegend; 1:200, clone: 93) were added and samples were incubated for 10 minutes at room temperature. 25 μL of a master mix of the desired surface marker antibodies (CD8.2b: Pacific Blue clone 53–5.8; CD4: FITC, clone H129.19; CD4: PE, clone GK1.5; CD3: APC, clone 145-2C11; CD44: BV605, clone IM7; CD62L: AF700, clone MEL-14; CD19: APC-Cy7, clone 6D5; all Biolegend; 1:200 in PBS) and Fixable Viability Dye eFluor 780 (eBioscience; 1:2000 in PBS) were added, followed by a 20 minutes incubation at 4°C. Cells were washed with FACS buffer (PBS, 2% FCS) and fixed in 4% Paraformaldehyde/PBS (Sigma) for 10 minutes at room temperature. Samples were washed twice with FACS buffer, resuspended in 100 μL and analyzed by flow cytometry on a BD LSRFortessa.

For intracellular cytokine staining (ICS), after plating, cell pellets were resuspended in 50 μL of RPMI 1640 medium (Gibco) supplemented with 10% FCS (PAA) and 1% Penicillin-Streptomycin-Glutamine (Thermo Fisher Scientific), 50 μM β-mercaptoethanol (Sigma), containing LCMV peptides (1:1000, Peptide 2.0 Inc.) and Protein Transport Inhibitor Cocktail (eBioscience, #00-4980-03; 1:500, Thermo Fisher Scientific). As positive control, cells were treated with Cell Stimulation Cocktail (eBioscience, #00-4970-93). Cells were incubated for 4 hours at 37°C and surface antigens were stained as described above. Afterwards, a master mix of desired antibodies in 25 μL FACS buffer containing 0.05% saponin (Sigma, 47036) against intracellular antigens of interest were added and incubated for 90 minutes at 4°C (IFNγ: PE-Cy7, clone XMG1.2; IL-2: PE, clone JES6-5H4; TNFα: APC, clone MP6-XT22; all Biolegend. all 1:200). Lastly, cells were washed twice with FACS buffer, resuspended in 100 μL and analyzed by flow cytometry.

### RNA sequencing

RNA quality was assessed via an Experion RNA Highsense chip (Biorad) and the RNA sequencing (RNA-seq) libraries were prepared using the TruSeq RNA sample preparation kti v2 (Illumina) according to the manufacturer’s instructions. All cDNA library samples were quantified, and quality controlled using a Qubit Fluorometric assay (Life Tech) and an Experion DNA Analysis chip (Biorad), respectively. 15 samples per lane were multiplexed and run on a 50 bp single end flow cell on a HiSeq3000 sequencing instrument (Illumina). Bases were called bases via the Illumina Realtime Analysis software and converted into BAM format using Illumina2bam and demultiplexed using BamIndexDecoder (https://github.com/wtsi-npg/illumina2bam)). The RNA-seq analysis pipeline used Tuxedo and reads were mapped on the mouse reference genome (Mus musculus, Ensembl e87, December 2016) using TopHat2 (v2.0.10). Transcripts were assembled from spliced read alignments via Cufflinks (v2.2.1), using the Ensembl e87 transcriptome as the reference as well as *de novo* assembly of transcript models. Differential expression analysis was quantified with Cuffdiff (v2.2.1). Transcriptome sets of all replicates for each sample group were combined with Cuffmerge and expression values were reported as FPKM (fragments per kilobase pf transcript per million). Identification of differentially regulated genes between conditions was based on an expression level cutoff of ≥ 1 FPKM, adjusted p value ≤ 0.05 and absolute log2 fold-change of 0.7.

### Enrichments and pathway analyses

Enrichment analyses on the union of differential modulated entities (transcripts and/or proteins) specific clusters were done in Cytoscape ClueGO [29] v2.5.6, based on GO (Immune System Process), KEGG and Reactome pathways. Terms were called enriched based on a maximum p-value of 0.05 and a minimum of 4% gene overlap. GO Term Fusion and grouping was applied. Enriched groups where further ranked according to the group Bonferroni step-down adjusted p-value.

### Targeted LC-MS based metabolite measurements

Per mg tissue, 3 μL of 80% (v/v) methanol were added and the tissue was lyzed using a Precellys 24 tissue homogenizer (Precellys CK14 lysing kit, Bertin). 10 μL of the resulting homogenate or serum were mixed with 10 μL of an isotopically labeled internal standard mixture in a hydrophobic 96well filter plate. 300 μL of methanol were added and mixed for 20 min at 450 rpm. The filter plates were centrifuging 5 min at 500 g to collect the extracts. LC-MS analysis was performed on a Vanquish UHPLC system (Thermo Scientific) coupled to an Orbitrap Q Exactive (Thermo Scientific) mass spectrometer. The chromatographic separation for samples was done on a ACQUITY UPLC BEH Amide, 1.7 μm, 2.1x100 mm analytical column (Waters) equipped with a VanGuard: BEH C18, 2.1x5mm pre-column (Waters). The column was maintained at a temperature of 40°C and 2 μL sample were injected per run. The mobile phase A was 0.15% formic acid (v/v) in water and mobile phase B was 0.15% formic acid (v/v) in 85% acetonitrile (v/v) with 10 mM ammonium formate. Total analysis time was 17 min at a flow rate 0.4 mL/min. The Orbitrap Q Exactive (Thermo Scientific) mass spectrometer was operated in an electrospray ionization positive mode, spray voltage 3.5 kV, aux gas heater temperature 400°C, capillary temperature 350°C, aux gas flow rate 12. Metabolites of interest were detected in full MS scan mode, with a scan range m/z 50 to 400, resolution 35000, AGC target 1e6 and maximum IT 50ms. Data processing was done using the Trace Finder 4.1 software (Thermo Scientific). Seven-point linear calibration curves with internal standardization and 1/x weighing was constructed for the quantification of metabolites.

### Statistical information

Data is presented as arithmetic mean ± standard error of the mean (S.E.M.). Statistical significances were calculated using a Student’s t-test when comparing two groups or using two-way ANOVA with Bonferroni correction for time-series. * P < 0.05 ** P < 0.01 *** P < 0.001.

## Supporting information

S1 Fig Enrichment analyses of liver-associated and interferon stimulated genes (ISGs) upon type I interferon treatment of primary hepatocytes. (A) Enrichment of liver-enriched and interferon stimulated genes (ISGs) in primary murine hepatocytes 24h after IFNβ treatment. Number of significantly regulated liver genes or ISGs is normalized to total detected liver genes or ISGs normalized to the ratio of total significant genes to total detected genes.
(TIF)

S2 Fig Hepatocyte-intrinsic type I interferon signaling specifically induces *Tdo2*, but not *Ido2* expression. (A) *Ido2* transcript levels in liver tissue of hepatitis C virus infected patients upon treatment with pegylated type I interferon. (B) *Tdo2* expression levels in naïve and LCMV clone 13 infected *Alb-Cre ERT2 Ifnar1*^*fl/fl*^ (*Ifnar1*^*Δ/Δ*^) and *Ifnar1*^*+/+*^ mice (n = 3–5) measured at the indicated time points via RT-qPCR. Symbols represent the arithmetic mean ±S.E.M. ns = not significant * P < 0.05 ** P < 0.01 *** P < 0.001 (Student’s t-test).
(TIF)

S3 Fig Viral infection and type I interferon induce *Tdo2* in hepatocytes but not *Ido1* or *Ido2*. (A-D) UMAP plots showing expression levels of *Tdo2*, *Ido1* and *Ido2* and (E) violin plot showing expression levels of *Tdo2* in hepatocytes isolated from naïve versus LCMV clone 13 infected (2 days after infection) wild type (C57Bl/6J) animals. Each dot represents a single hepatocyte. (F) *Tdo2*, *Ido1* and *Ido2* transcript levels in liver tissue of LCMV clone 13 infected (2 and 8 days after infection) wild type (C57Bl/6J, n = 3) or (G) LCMV clone 13 infected (1.5 days after infection) *Alb-Cre ERT2 Ifnar1*^*fl/fl*^ (*Ifnar1*^*Δ/Δ*^) and *Ifnar1*^*+/+*^ mice (n = 3) measured via RNA sequencing. (H) *Tdo2*, *Ido1* and *Ido2* transcript levels in primary murine hepatocytes 24h after IFNβ treatment measured via RNA sequencing. (I) IDO1 and (J) IDO2 liver protein levels determined via quantitative proteomics of LCMV Cl13 infected wild type mice at 0, 2 and 8dpi (n = 2). Symbols represent the arithmetic mean ±S.E.M. ns = not significant * P < 0.05 ** P < 0.01 *** P < 0.001 (Student’s t-test).
(TIF)

S4 Fig *Tdo2*-deficiency does not affect innate immune responses and *Ido1* expression in the spleen upon LCMV infection. (A) Viral load in blood, liver, spleen and kidney of LCMV clone 13 infected (2 days after infection) *Tdo2*-deficient animals compared to littermate wild type animals (n = 5–7) measured by focus forming assay. (B) *Ifit1* expression levels in liver tissue after LCMV Cl13 clone 13 infected (2 days after infection) (n = 3–4) measured by RT-qPCR. (C) Serum IFNα levels after LCMV clone 13 infection (2 days after infection, n = 3–5). (D) Abundance of CD4 and CD8 splenic T cells and PD1^+^ and (E-F) CD44^+^ and CD62L^+^ expressing splenic T cells in naive *Tdo2*-deficient animals compared to wild type animals. (G) Abundance of CD4 and CD8 splenic T cells after LCMV clone 13 infection (8 days after infection, n = 3–5). (H) GP33- and NP396-specific CD8 T cells after LCMV clone 13 infection (8 days after infection, n = 3–5). (I-K) IFNγ and TNFα production of LCMV GP33-, NP396- and GP276-specific splenic CD8 T cells after LCMV clone 13 infection (50 days after infection) (n = 5). (L) Serum albumin levels of LCMV clone 13 infected Tdo2-deficient animals compared to littermate wild type animals (n = 5). (M-O) Viremia and RNemia blood, liver, spleen and kidney of LCMV clone 13 infected (8 and 50 days after infection) *Tdo2*-deficient animals compared to littermate wild type animals (n = 5–7) measured by focus forming assay and RT-qPCR. (P) Serum tryptophan to kynurenine ratio after LCMV clone 13 infection (8 days after infection) of *Tdo2*-deficient animals compared to littermate wild type animals (n = 3–5). Symbols represent the arithmetic mean ±S.E.M. ns = not significant * P < 0.05 ** P < 0.01 *** P < 0.001 (Student’s t-test).
(TIF)

S5 Fig *Tdo2* expression in primary hepatocytes and IDO1 and IDO2 levels and activity upon *Tdo2*-deficiency. (A) *Tdo2* gene expression levels in primary hepatocytes upon IFNβ (1000 U/mL), L-tryptophan (1 mM) or LCMV clone 13 infection (MOI 3). (B) *Ido1* and (C) *Ido2* transcript levels in liver spleen and kidney tissue of naive or LCMV clone 13 infected (8 days after infected) *Tdo2*-deficient and wild type littermate control animals (n = 3–5). (D) L-kynurenine concentations in cell culture supernatants of primary *Tdo2*-deficient and wild type primary hepatocytes upon 1-methyl-L-tryptophan (1-MT, 1 mM) or the Epacadostat (500 nM) (n = 3) treatment. (E) Representative western blot of IDO1 from lung (n = 1, positive control) and liver tissue (n = 2) of LCMV clone 13 infected (8days after infection) *Tdo2*-deficient and wild type littermate controls. Symbols represent the arithmetic mean ±S.E.M. ns = not significant * P < 0.05 ** P < 0.01 *** P < 0.001 (Student’s t-test).
(TIF)
