# Supplementary figures and images for: Hepatocyte-intrinsic type I interferon signaling reprograms metabolism and reveals a novel compensatory mechanism of the tryptophan-kynurenine pathway in viral hepatitis
Source: PLoS Pathog. 2020 Oct 12;16(10):e1008973. doi: 10.1371/journal.ppat.1008973 (PMC7580883; doi:10.1371/journal.ppat.1008973)

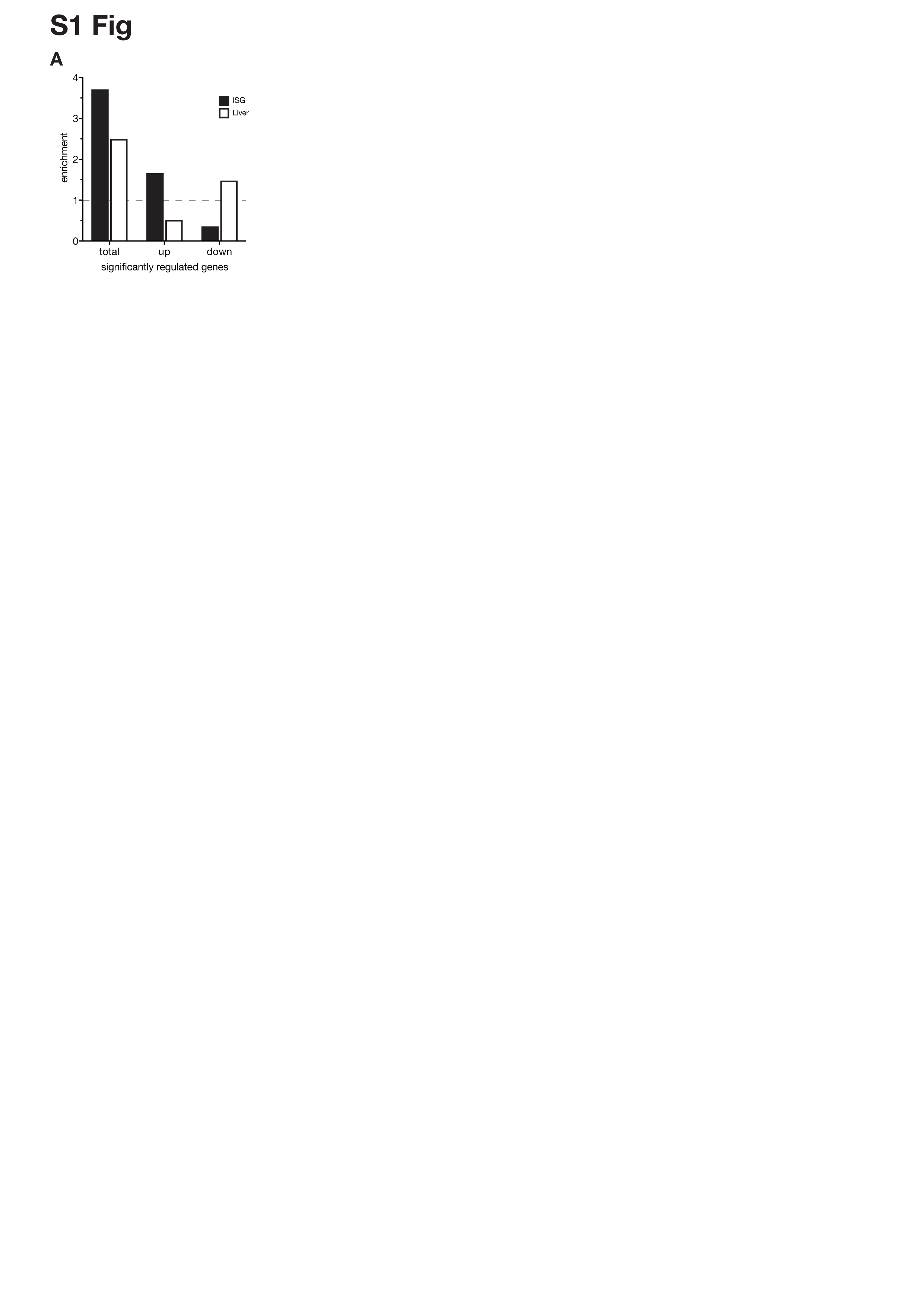

Supplement: S1 Fig — (A) Enrichment of liver-enriched and interferon stimulated genes (ISGs) in primary murine hepatocytes 24h after IFNβ treatment. Number of significantly regulated liver genes or ISGs is normalized to total detected liver genes or ISGs normalized to the ratio of total significant genes to total detected genes. (TIF) [file ppat.1008973.s001.tif]

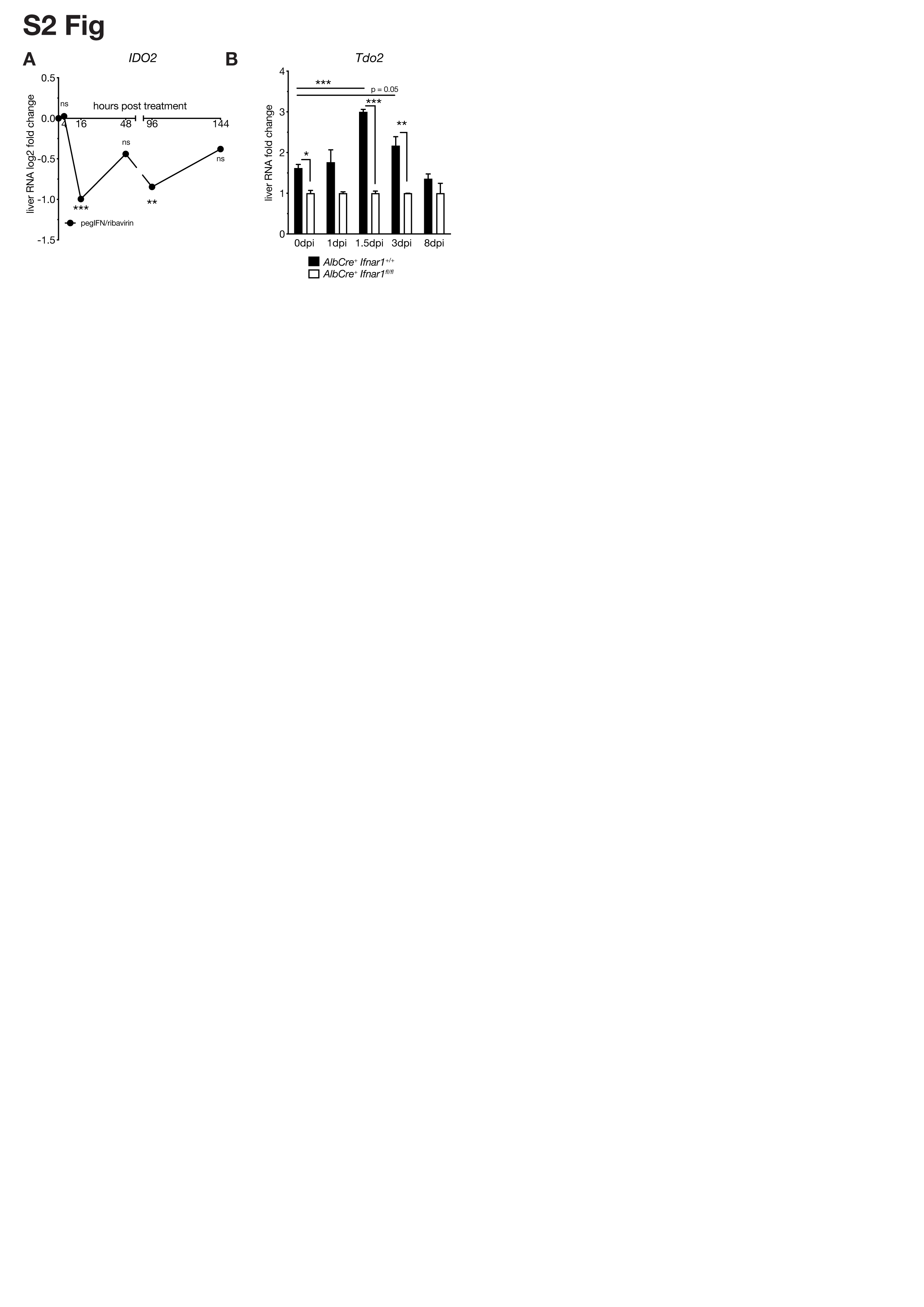

Supplement: S2 Fig — (A) Ido2 transcript levels in liver tissue of hepatitis C virus infected patients upon treatment with pegylated type I interferon. (B) Tdo2 expression levels in naïve and LCMV clone 13 infected Alb-Cre ERT2 Ifnar1fl/fl (Ifnar1Δ/Δ) and Ifnar1+/+ mice (n = 3–5) measured at the indicated time points via RT-qPCR. Symbols represent the arithmetic mean ±S.E.M. ns = not significant * P < 0.05 ** P < 0.01 *** P < 0.001 (Student’s t-test). (TIF) [file ppat.1008973.s002.tif]

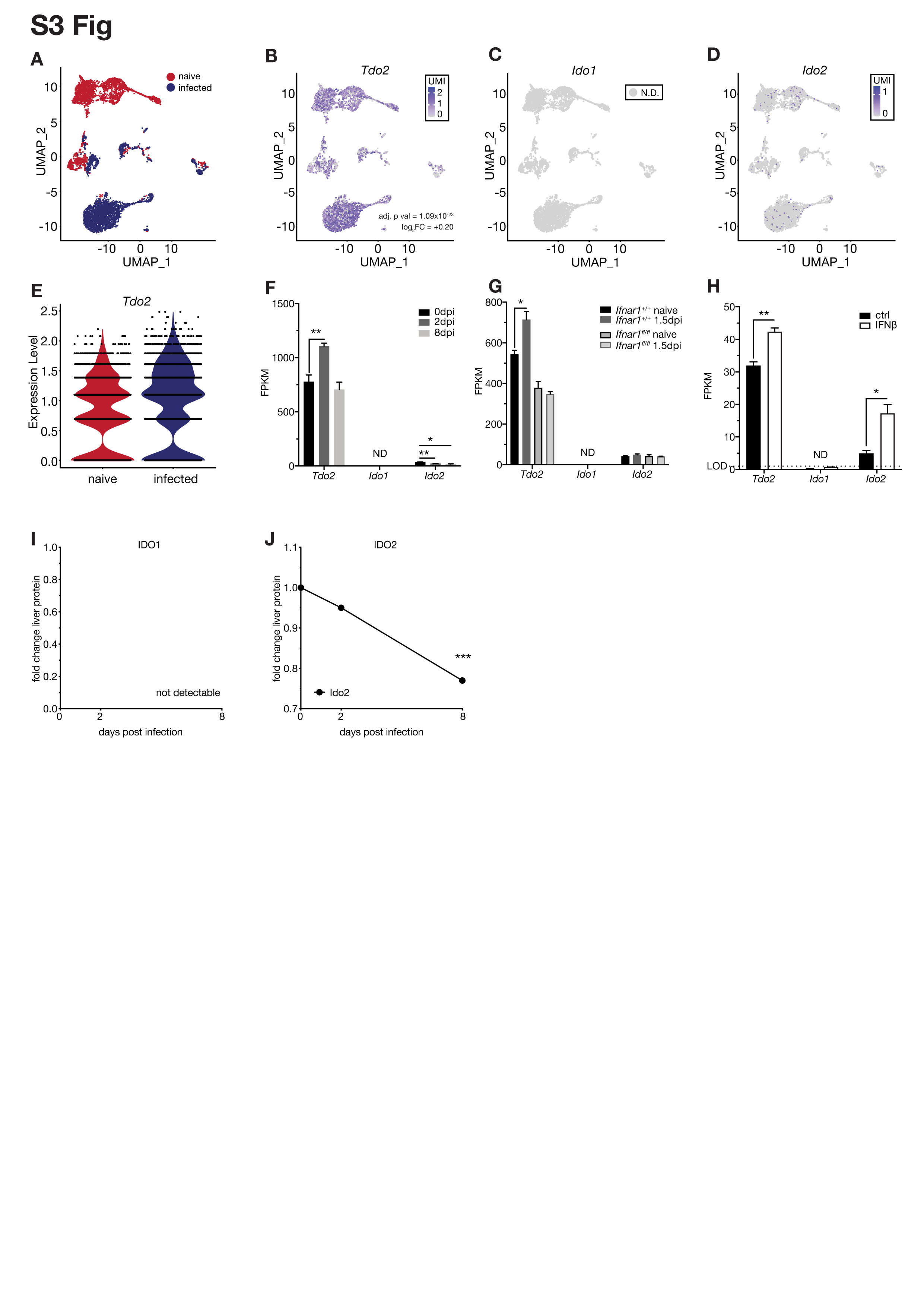

Supplement: S3 Fig — (A-D) UMAP plots showing expression levels of Tdo2, Ido1 and Ido2 and (E) violin plot showing expression levels of Tdo2 in hepatocytes isolated from naïve versus LCMV clone 13 infected (2 days after infection) wild type (C57Bl/6J) animals. Each dot represents a single hepatocyte. (F) Tdo2, Ido1 and Ido2 transcript levels in liver tissue of LCMV clone 13 infected (2 and 8 days after infection) wild type (C57Bl/6J, n = 3) or (G) LCMV clone 13 infected (1.5 days after infection) Alb-Cre ERT2 Ifnar1fl/fl (Ifnar1Δ/Δ) and Ifnar1+/+ mice (n = 3) measured via RNA sequencing. (H) Tdo2, Ido1 and Ido2 transcript levels in primary murine hepatocytes 24h after IFNβ treatment measured via RNA sequencing. (I) IDO1 and (J) IDO2 liver protein levels determined via quantitative proteomics of LCMV Cl13 infected wild type mice at 0, 2 and 8dpi (n = 2). Symbols represent the arithmetic mean ±S.E.M. ns = not significant * P < 0.05 ** P < 0.01 *** P < 0.001 (Student’s t-test). (TIF) [file ppat.1008973.s003.tif]

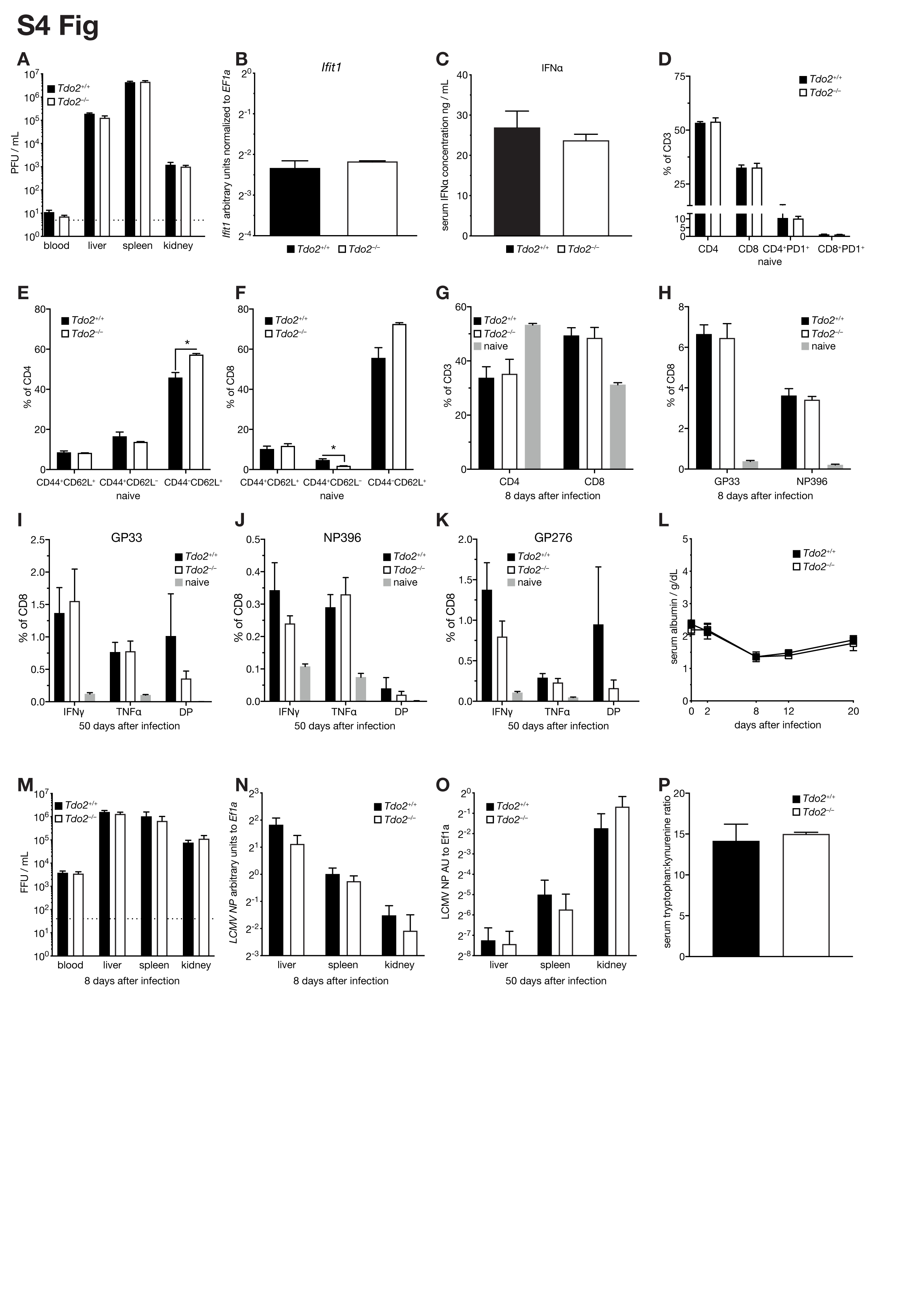

Supplement: S4 Fig — (A) Viral load in blood, liver, spleen and kidney of LCMV clone 13 infected (2 days after infection) Tdo2-deficient animals compared to littermate wild type animals (n = 5–7) measured by focus forming assay. (B) Ifit1 expression levels in liver tissue after LCMV Cl13 clone 13 infected (2 days after infection) (n = 3–4) measured by RT-qPCR. (C) Serum IFNα levels after LCMV clone 13 infection (2 days after infection, n = 3–5). (D) Abundance of CD4 and CD8 splenic T cells and PD1+ and (E-F) CD44+ and CD62L+ expressing splenic T cells in naive Tdo2-deficient animals compared to wild type animals. (G) Abundance of CD4 and CD8 splenic T cells after LCMV clone 13 infection (8 days after infection, n = 3–5). (H) GP33- and NP396-specific CD8 T cells after LCMV clone 13 infection (8 days after infection, n = 3–5). (I-K) IFNγ and TNFα production of LCMV GP33-, NP396- and GP276-specific splenic CD8 T cells after LCMV clone 13 infection (50 days after infection) (n = 5). (L) Serum albumin levels of LCMV clone 13 infected Tdo2-deficient animals compared to littermate wild type animals (n = 5). (M-O) Viremia and RNemia blood, liver, spleen and kidney of LCMV clone 13 infected (8 and 50 days after infection) Tdo2-deficient animals compared to littermate wild type animals (n = 5–7) measured by focus forming assay and RT-qPCR. (P) Serum tryptophan to kynurenine ratio after LCMV clone 13 infection (8 days after infection) of Tdo2-deficient animals compared to littermate wild type animals (n = 3–5). Symbols represent the arithmetic mean ±S.E.M. ns = not significant * P < 0.05 ** P < 0.01 *** P < 0.001 (Student’s t-test). (TIF) [file ppat.1008973.s004.tif]

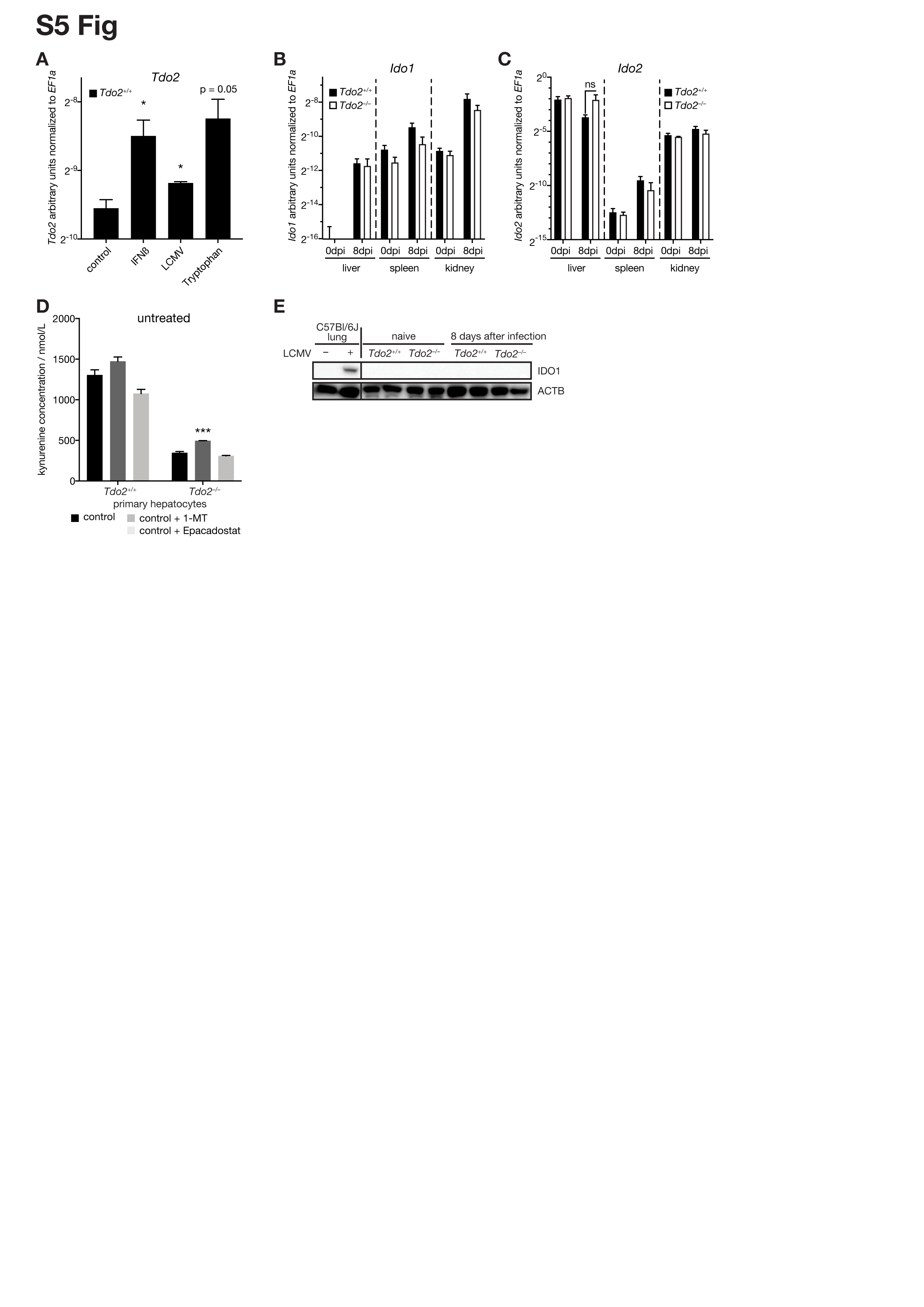

Supplement: S5 Fig — (A) Tdo2 gene expression levels in primary hepatocytes upon IFNβ (1000 U/mL), L-tryptophan (1 mM) or LCMV clone 13 infection (MOI 3). (B) Ido1 and (C) Ido2 transcript levels in liver spleen and kidney tissue of naive or LCMV clone 13 infected (8 days after infected) Tdo2-deficient and wild type littermate control animals (n = 3–5). (D) L-kynurenine concentations in cell culture supernatants of primary Tdo2-deficient and wild type primary hepatocytes upon 1-methyl-L-tryptophan (1-MT, 1 mM) or the Epacadostat (500 nM) (n = 3) treatment. (E) Representative western blot of IDO1 from lung (n = 1, positive control) and liver tissue (n = 2) of LCMV clone 13 infected (8days after infection) Tdo2-deficient and wild type littermate controls. Symbols represent the arithmetic mean ±S.E.M. ns = not significant * P < 0.05 ** P < 0.01 *** P < 0.001 (Student’s t-test). (TIF) [file ppat.1008973.s005.tif]
